# Cholesteryl ester transfer protein *Taq*IB polymorphism and its association with serum lipid levels and longevity in Chinese Bama Zhuang population

**DOI:** 10.1186/1476-511X-11-26

**Published:** 2012-02-15

**Authors:** Shang-Ling Pan, Fei Wang, Ze-Ping Lu, Cheng-Wu Liu, Cai-You Hu, Huan Luo, Jun-Hua Peng, Xiao-Qiu Luo, Guo-Fang Pang, Shao-Hua Lu, Hua-Yu Wu, Ling-Jin Huang, Rui-Xing Yin

**Affiliations:** 1 Department of Cardiology, Institute of Cardiovascular Diseases, the First Affiliated Hospital, Guangxi Medical University, 22 Shuangyong Road, Nanning 530021, Guangxi, People's Republic of China; 2 Department of Pathophysiology, School of Premedical Sciences, Guangxi Medical University, Nanning 530021, Guangxi, People's Republic of China; 3 Department of Neurology, Jiangbin Hospital of Guangxi Zhuang Autonomous Region, 85 Hedi Road, Nanning 530021, Guangxi, People's Republic of China

## Abstract

**Background:**

*Taq*IB polymorphism in the cholesteryl ester transfer protein (CETP) gene has been reported to be associated with serum high-density lipoprotein cholesterol (HDL-C) levels and longevity in several populations, but controversial results also arose probably due to racial/ethnic diversity. Bama is a remote and mountainous county located in the northwest of Guangxi, People's Republic of China, which has been well known for its longevity for centuries. The current study was to investigate the possible association of CETP *Taq*IB polymorphism with serum lipid levels and longevity in the Bama Zhuang population.

**Methods:**

The CETP *Taq*IB genotypes were determined by polymerase chain reaction and restriction fragment length polymorphism in 523 long-lived inhabitants (long-lived group, LG; aged 90-107 years) and 498 healthy controls without longevity family history (non-long-lived group, non-LG; aged 40-69 years) residing in Bama County.

**Results:**

The levels of total cholesterol (TC), high-density lipoprotein cholesterol (HDL-C), and low-density lipoprotein cholesterol (LDL-C) were higher but TG, HDL-C/LDL-C ratio and the prevalence of dyslipidemia were lower in LG than in non-LG (*P *< 0.001 for all). There were no differences in the allelic and genotypic frequencies between the two groups (*P *> 0.05). Serum HDL-C levels and HDL-C/LDL-C ratio in LG were different among the genotypes (*P *< 0.01 for each), the subjects with B2B2 and B1B2 genotyes had higher HDL-C levels and HDL-C/LDL-C ratio than the subjects with B1B1genotye, whereas the levels of TC and HDL-C in non-LG were different among/between the genotypes (*P *< 0.01 for each), the B2 allele carriers had lower TC and higher HDL-C levels than the B2 allele noncarriers. Serum TG and HDL-C levels and HDL-C/LDL-C ratio were correlated with genotypes in LG, whereas serum TC and HDL-C levels were associated with genotypes in non-LG (*P *< 0.05-0.001).

**Conclusions:**

The association of CETP *Taq*IB polymorphism and serum lipid profiles is different between LG and non-LG in the Chinese Bama Zhuang population. CETP *Taq*IB polymorphism might be one of the longevity-related genetic factors in this population.

## Introduction

Cholesterol plays an essential physiological role in humans. It is mainly synthesized and esterified by the liver and then is secreted from the liver into plasma as very low density lipoprotein (VLDL), which gets converted to low density lipoprotein (LDL). In contrast, reverse cholesterol transport (RCT) mediates the conveyance of free unesterified cholesterol mobilized from peripheral cells and destined for disposal by the liver. In this process, cholesteryl ester transfer protein (CETP), a 74-kDa hydrophobic glycoprotein, facilitates the transfer of cholesterol ester from high density lipoprotein (HDL) to apolipoprotein (Apo) B-containing lipoproteins including VLDL and LDL, for disposal via the LDL receptor pathway in the liver, and of triglyceride (TG) in the opposite direction in plasma [1,2]. Remarkably, these rationales indicate that CETP plays a significant role in the modulation of cholesterol.

In humans, CETP is expressed predominantly in the liver, spleen, and adipose tissue [1]. Detectable levels of CETP can also be seen in the small intestine, adrenal glands, heart, kidneys, or skeletal muscle [3]. The gene encoding CETP consists of 16 exons and 15 introns encompassing 25 kb on chromosome 16q12-21 adjacent to the lecithin-cholesterol acyltransferase gene [4]. To date, a number of common polymorphisms and rare variants at the CETP gene locus, such as I405V, D442G, I14A, A373P, R451Q, promoter polymorphism (Y629A/C, Y1337C/T and Y971G/A), which cause depletion of CETP activity and consequently high high-density lipoprotein cholesterol (HDL-C) in plasma, have been described across populations [5-8]. One of these common polymorphisms is *Taq*IB, a silent base change affecting the 277th nucleotide in the first intron of the CETP gene [4]. The B2 allele, absence of the *Taq*I restriction site, has been found to be associated with elevated plasma HDL-C level and reduced plasma CETP mass and activity and coronary heart disease (CHD) risk [9-12], and accordingly to be associated with longer life expectancy [13,14]. This hypothesis is further supported by the fact that high HDL-C levels are often observed in healthy elderly aged 85 and above [13,15]. However, inconsistent findings also arose in different studies, e.g., a positive association of HDL-C levels with increased CHD risk has been reported [16]; not all CETP deficiencies with high HDL-C levels are correlated with longevity [17]. These controversial results suggest that the roles of CETP gene variations in longevity appear much more complex than expected and need further elucidation.

Bama is a remote and mountainous county located in the northwest of Guangxi, People's Republic of China. It has been well known for its longevity for centuries. The population size and centenarian rate in Bama County are around 240,000 and 30/100,000 respectively, according to the National Population Census of China in the past decades [18,19]. Although a number of studies involving natural environment, dietary habit, socioeconomic status and genetic background have been conducted for decades [19-22], the underlying mechanisms of the longevity in this county are still unknown. Genetically, the majority (> 85%) of the inhabitants living in Bama County belong to the north branch of Zhuang ethnic group. Zhuang is the largest minority in China with a total population of 15 millions [23,24]. Long-term geographic and social isolation as well as inbreeding result in both cultural and genetic homogeneity of Bama Zhuang branch, and therefore Bama Zhuang has become a useful subgroup for population genetic studies. We have initiated the Bama Longevity Genetic Study (BLGS) since late 1998. Several genetic polymorphisms such as human leukocyte antigen (HLA), ApoE, p53, and transforming growth factor-β1 (TGF-β1) genes and the haplotypes of mitochondria DNA [19,25-29] have been shown difference between long-lived group (LG) and non-long-lived group (non-LG), indicating that Bama long-lived individuals may have favorable genetic background for their survivals. Therefore, the aim of the present study was to determine the *Taq*IB polymorphism of CETP gene and its association with serum lipid levels and longevity in the Chinese Bama Zhuang population.

## Materials and methods

### Study population

A total of 523 long-lived subjects (339 females and 184 males, LG) with exceptional longevity residing in Bama County, Guangxi, People's Republic of China were recruited to participate in the study, which was conducted from 2008 to 2011. The ages of the subjects ranged from 90 to 107 years, with an average age of 93.38 ± 3.09 years. The ages were defined by dates of birth as stated on identity cards. During the same period, a total of 498 healthy participants (253 males and 245 females, non-LG) without longevity family history were also randomly selected from the same area. The average age of the participants was 53.12 ± 8.86 years (range, 40-69 years). All study subjects were unrelated and belong to Zhuang ethnic group. They have been living in Bama rural area and doing farm work lifelong. All subjects were essentially healthy and had no evidence of any chronic illness, including hepatic, renal, or thyroid. The participants with a history of myocardial infarction, stroke, diabetes were also excluded. The participants were not taking medications known to affect serum lipid levels such as statins or fibrates, beta-blockers, diuretics, or hormones. The current study was approved by the Ethics Committee of Guangxi Medical University. Informed consent was obtained from all subjects after they received a full explanation of the study.

### Epidemiological survey

Information on demography and lifestyle factors was collected with standardized questionnaires. The physical examination included blood pressure, body height, body weight, waist circumference, and body mass index (BMI) was calculated as weight in kilograms divided by the square of height in meters (kg/m^2^). Sitting blood pressure was obtained 3 times, using a standard mercury sphygmomanometer with the subject resting for at least 5 minutes, and the average of the 3 measurements was used for the level of blood pressure. Systolic blood pressure was determined by the first Korotkoff sound; and diastolic blood pressure, by the fifth Korotkoff sound. Hypertension was defined as an average systolic blood pressure of 140 mmHg or greater and an average diastolic blood pressure of 90 mmHg or greater, and/or self-reported pharmacological treatment for hypertension within the 2 weeks prior to the interview [30]. Normal weight, overweight, and obesity were defined as BMI < 24, 24 to 28, and > 28 kg/m^2^, respectively [31].

### Biochemical analysis

A venous blood sample of 8 mL was drawn from each subject after an overnight fasting. 4 mL of the sample was collected in a glass tube for serum lipid determination. The remaining sample was transferred to a tube with anticoagulant solution (4.80 g/L citric acid, 14.70 g/L glucose, and 13.20 g/L trisodium citrate) for DNA extraction. The levels of serum total cholesterol (TC), TG, HDL-C, and LDL-C in samples were determined by enzymatic methods with commercially available kits, Tcho-1, TGLH (Randox Laboratories Ltd, Crumlin, Antrim, United Kingdom), Cholestest N HDL, and Cholestest LDL (Daiichi Pure Chemicals Co, Ltd., Tokyo, Japan), respectively. All determinations were performed by standard automated methods with a biochemical analyzer (Type 7170A; Hitachi Ltd, Tokyo, Japan) at the Clinical Science Experiment Center of the First Affiliated Hospital, Guangxi Medical University. The normal ranges of serum TC, TG, HDL-C, and LDL-C levels in the Center were 3.10-5.17, 0.56-1.70, 0.91-1.81, and 1.70-3.20 mmol/L, respectively. The individuals with TC > 5.17 mmol/L and/or TG > 1.70 mmol/L were defined as hyperlipidemic [32].

### DNA amplification and genotyping

Genomic DNA was isolated from peripheral blood leukocytes by standard methods [33]. Genotyping of the CETP *Taq*IB was performed as described previously [34] basing on the protocol introduced by Fumeron *et al. *[35]. Briefly, a 535 bp fragment in intron 1 of the CETP gene was amplified by polymerase chain reaction (PCR), with use of the following primers: F: 5'-CACTAGCCCAGAGAGAGGAGTGCC-3' and R: 5'-CTGAGCCCAGCCGCACACTAA-3'. (Sangon Biotech Co., Ltd., Shanghai, People's Republic of China). PCR was performed in a volume of 20 μL containing 200 ng of genomic DNA, plus 10 μL of *Taq *MasterMix (Beijing CoWin Bioscience Co., Ltd. People's Republic of China), 6.25 μM (1.0 μL) of each primer, 7 μL ddH_2_O and 1 U of DNA polymerase (Takara Biotechnology Co, Ltd, Dalian, People's Republic of China). The cycle profile was as follows: predenaturation at 94°C for 2 min, followed by 30 cycles of denaturation at 94°C for 30 s, annealing at 63°C for 30 s, and extension at 72°C for 30 s, with a final extension at 72°C for 5 min. The PCR products (8 μL) were digested with *Taq*I (0.2 U) restriction endonuclease (Takara Biotechnology Co, Ltd, Dalian, People's Republic of China) at 65°C for 4 h, and the fragments were separated by electrophoresis in a 2% agarose gel for 45 min at 80 V. The target DNA fragments were 174- and 361-bp for the B1 allele and 535 bp for the B2 allele. The genotypes were identified and named according to the presence or absence of the enzymatic restriction sites, i.e. bands at 535-, 361-, and 174-bp for B1B2 heterozygotic genotype, 361- and 174-bp for B1B1 homozygotic genotype, and 535 bp for B2B2 homozygotic genotype. Laboratory technicians were masked to clinical and biochemical data.

### Statistical analyses

Levels of the quantitative variables are presented as mean ± SD (serum TG levels are presented as medians and interquartile ranges). Hardy-Weinberg equilibrium was computed for the expected genotype distribution. Allelic and genotypic frequencies were calculated directly. Comparison of mean values of general characteristics between study groups was performed with the student unpaired *t *test and One-way ANOVA test. The statistical evaluation for the categorical variables was based on the calculation of the chi-square and Fisher's exact criteria. The association of CETP *Taq*IB genotypes with serum lipid variables was tested by analysis of covariance. In order to assess the association of serum lipid levels with genotypes (B1B1 = 1, B1B2 = 2 and B2B2 = 3), multivariable linear regression analyses with stepwise modeling were also performed in the combined population of LG and non-LG, LG, non-LG; respectively. All tests were two-sided and *P*-values of 0.05 were considered significant. Data were analyzed using the statistical software package SPSS 13.0 (SPSS Inc, Chicago, IL).

## Results

### General characteristics and serum lipid levels

A summary of demographic, clinical, and biochemical characteristics of LG and non-LG is provided in Table 1. BMI was lower in LG as compared to non-LG (*P *< 0.001). The levels of systolic blood pressure and diastolic blood pressure were significantly higher in LG than in non-LG (*P *< 0.001 for all). The levels of TC, HDL-C, and LDL-C were markedly higher, while TG, HDL-C/LDL-C ratio as well as the prevalence of dyslipidemia were lower in LG as compared with non-LG (*P *< 0.001 for all).

**Table 1:** Clinical characteristics and serum lipid profiles between LG and non-LG

Paramenter	LG (n = 523)	non-LG (n = 498)	*t *(*χ^2^*)	*P*
Age (years)	93.38 ± 3.06	53.12 ± 8.86	96.088	0.000
Gender (M/F)	184/339	253/245	25.428	0.000
Body mass index (kg/m^2^)	20.22 ± 3.52	22.14 ± 3.67	-8.521	0.000
Systolic blood pressure (mmHg)	166.14 ± 28.04	131.31 ± 20.23	22.844	0.000
Diastolic blood pressure (mmHg)	89.33 ± 13.48	85.24 ± 11.43	5.240	0.000
Total cholesterol (mmol/L)	5.13 ± 1.01	4.87 ± 0.96	4.177	0.000
Triglyceride (mmol/L)	0.97 (0.47)	1.08 (0.86)	-3.256	0.001
HDL-C (mmol/L)	1.60 ± 0.35	1.51 ± 0.29	4.698	0.000
LDL-C (mmol/L)	3.04 ± 0.87	2.69 ± 0.89	6.427	0.000
HDL-C/LDL-C ratio	0.57 ± 0.24	0.67 ± 0.59	-3.466	0.001
Dyslipidemia [n (%)]	66(12.62)	94(18.88)	-7.555	0.000

HDL-C, high-density lipoprotein cholesterol; LDL-C, low-density lipoprotein cholesterol. The value of triglyceride was presented as median (interquartile range), the difference between the two groups was determined by the Wilcoxon-Mann-Whitney test.

### Genotypic and allelic frequencies

The CETP *Taq*IB genotype and allele frequencies are shown in Table 2. The dominant allele was B1, and the dominant genotypes were B1B2 and B1B1 in both groups. Homozygotic B2B2 genotype only accounted for approximately 10% in the studied participants. No significant difference was observed in the distribution of genotypes and alleles between the two groups (both *P *> 0.05), albeit the frequency of B2 allele was slightly higher in the longevity group as compared with the counterparts.

**Table 2:** Genotype and allele frequencies of the CETP *Taq*IB polymorphism in LG and non-LG [n (%)]

Groups	n	Genotype			Allele	
				
		B1B1	B1B2	B2B2	B1	B2
LG	523	187 (35.8)	270 (51.6)	66 (12.6)	644 (61.6)	402 (38.4)
Non-LG	498	206 (41.4)	240 (48.2)	52 (10.4)	652 (65.5)	344 (34.5)
*χ*^2^	-	3.734			3.336	
*P*	-	0.155			0.068	

### Genotypes and serum lipid levels

As shown in Table 3, the levels of HDL-C and the ratio of HDL-C to LDL-C in LG were different among the genotypes (*P *< 0.01 for each), the subjects with B2B2 and B1B2 genotyes had higher HDL-C levels and HDL-C/LDL-C ratio than the subjects with B1B1genotye, whereas the levels of TC and HDL-C in non-LG were different among/between the genotypes (*P *< 0.01 for each), the B2 allele carriers had lower TC and higher HDL-C levels than the B2 allele noncarriers. When the association of CETP *Taq*IB polymorphism and serum lipid levels was stratified by gender, we found that serum TC, TG, and HDL-C levels in the combined population were different among the genotypes in males (*P *< 0.05-0.001), and HDL-C and LDL-C levels were different among the genotypes in females (*P *< 0.01 and *P *< 0.05; respectively; Table 4). Serum HDL-C levels in LG were different among the genotypes in males (*P *< 0.05) but not in females (Table 5). Serum HDL-C levels in non-LG were different among the genotypes in males (*P *< 0.001), and TC, HDL-C and LDL-C levels were different among the genotypes in females (*P *< 0.05-0.001; Table 6).

**Table 3:** Comparison of serum lipid levels among the genotypes between the two groups

Group	Genotype	n	TC (mmol/L)	TG (mmol/L)	HDL-C (mmol/L)	LDL-C (mmol/L)	HDL-C /LDL-C
LG	B1B1	187	5.16 ± 1.00	1.03(0.59)	1.54 ± 0.31^b^	3.11 ± 0.84^b^	0.53 ± 0.17
	B1B2	270	5.09 ± 1.02	0.95(0.44)	1.63 ± 0.37^a^	3.01 ± 0.89^b^	0.60 ± 0.26
	B2B2	66	5.17 ± 0.99	0.97(0.41)	1.67 ± 0.35^a^	2.99 ± 0.88^b^	0.62 ± 0.28
*F*	-	-	0.283	3.845	4.868	0.867	6.592
*P*	-	-	0.753	0.146	0.008	0.421	0.001
	B1B1	187	5.16 ± 1.00	1.03(0.59)	1.54 ± 0.31	3.11 ± 0.84	0.53 ± 0.17
	B1B2/B2B2	336	5.11 ± 1.01	0.95(0.43)	1.64 ± 0.37	3.00 ± 0.89	0.60 ± 0.27
*F*	-	-	0.247	-1.779	10.279	1.785	12.432
*P*	-	-	0.620	0.075	0.001	0.182	0.000
Non-LG	B1B1	206	4.79 ± 0.96	1.12(0.84)	1.45 ± 0.29	2.64 ± 0.90	0.70 ± 0.82
	B1B2	240	4.92 ± 0.93	1.04(0.84)	1.53 ± 0.28^a^	2.76 ± 0.90	0.64 ± 0.36
	B2B2	52	4.95 ± 1.09	1.27(1.11)	1.64 ± 0.28^a^	2.53 ± 0.81	0.74 ± 0.33
*F*	-	-	1.288	3.636	10.681	1.950	0.875
*P*	-	-	0.277	0.162	0.000	0.143	0.418
	B1B1	206	4.79 ± 0.96	1.12(0.84)	1.45 ± 0.29	2.64 ± 0.90	0.70 ± 0.82
	B1B2/B2B2	292	4.95 ± 0.96	1.06(0.88)	1.54 ± 0.29	2.73 ± 0.89	0.66 ± 0.36
*F*	-	-	4.713	-1.007	12.514	1.732	0.596
*P*	-	-	0.030	0.314	0.000	0.189	0.441

TC, total cholesterol; TG, triglyceride; HDL-C, high-density lipoprotein cholesterol; LDL-C, low-density lipoprotein cholesterol. The value of triglyceride was presented as median (interquartile range). The difference among/between the genotypes was determined by the Kruskal-Wallis test or Wilcoxon-Mann-Whitney test ^a ^*P *< 0.05 in comparison with B1B1 genotype of same group; ^b^*P *< 0.05 in comparison with the same genotype of non-LG

**Table 4:** Comparison of clinical characteristics and serum lipid levels among the genotypes stratified by gender in overall population

Parameter	B1B1	B1B2	B2B2	*F*	*P*
Male/n	166	224	47	-	-
Age (years)	70.15 ± 21.11	68.85 ± 21.37	69.62 ± 23.38	0.177	0.838
Body mass index (kg/m^2^)	21.48 ± 3.35	21.58 ± 3.43	21.17 ± 4.04	0.285	0.752
Systolic blood pressure (mmHg)	146.767 ± 26.80	142.51 ± 27.92	144.98 ± 26.29	1.169	0.312
Diastolic blood pressure (mmHg)	88.76 ± 12.64	85.90 ± 11.89	88.04 ± 14.55	2.615	0.074
Total cholesterol (mmol/L)	4.937 ± 1.09	4.88 ± 1.00	5.36 ± 1.12	4.237	0.015
Triglyceride (mmol/L)	1.08(0.67)	0.95(0.78)	1.18(0.74)	7.329	0.026
HDL-C (mmol/L)	1.47 ± 0.30	1.57 ± 0.33	1.71 ± 0.36	10.803	0.000
LDL-C (mmol/L)	2.85 ± 0.96	2.74 ± 0.94	2.86 ± 0.93	0.788	0.455
HDL-C/LDL-C	0.66 ± 0.89	0.67 ± 0.34	0.68 ± 0.33	0.010	0.990
Female/n	227	286	71	-	-
Age (years)	73.93 ± 21.91	78.76 ± 19.30	79.56 ± 18.62	4.249	0.015
Body mass index (kg/m^2^)	21.24 ± 4.52	20.47 ± 3.30	21.59 ± 3.70	3.777	0.023
Systolic blood pressure (mmHg)	150.42 ± 29.39	154.15 ± 33.03	154.17 ± 32.08	0.972	0.379
Diastolic blood pressure (mmHg)	88.18 ± 12.45	86.59 ± 13.09	88.35 ± 12.78	1.188	0.305
Total cholesterol (mmol/L)	4.99 ± 0.92	5.12 ± 0.96	4.88 ± 0.93	2.392	0.092
Triglyceride (mmol/L)	1.07(0.80)	1.00(0.49)	1.00(0.60)	2.739	0.254
HDL-C (mmol/L)	1.50 ± 0.30	1.59 ± 0.35	1.62 ± 0.29	5.739	0.003
LDL-C (mmol/L)	2.87 ± 0.85	3.02 ± 0.86	2.74 ± 0.84	3.810	0.023
HDL-C/LDL-C	0.57 ± 0.23	0.58 ± 0.29	0.66 ± 0.29	2.954	0.053

HDL-C, high-density lipoprotein cholesterol; LDL-C, low-density lipoprotein cholesterol. The value of triglyceride was presented as median (interquartile range), the difference among the genotypes was determined by the Kruskal-Wallis Test.

**Table 5:** Comparison of clinical characteristics and serum lipid Levels among genotypes stratified by gender in LG

Parameter	B1B1	B1B2	B2B2	*F*	*P*
Male/n	70	91	23	-	-
Age (years)	93.61 ± 3.43	93.17 ± 2.68	92.61 ± 2.73	1.081	0.341
Body mass index (kg/m^2^)	20.82 ± 3.70	20.30 ± 3.30	20.30 ± 5.05	0.426	0.654
Systolic blood pressure (mmHg)	164.79 ± 26.08	162.59 ± 27.50	161.22 ± 23.48	0.213	0.808
Diastolic blood pressure (mmHg)	88.60 ± 13.55	89.14 ± 13.73	88.70 ± 13.65	0.034	0.967
Total cholesterol (mmol/L)	4.97 ± 1.07	4.96 ± 1.09	5.30 ± 1.05	0.96	0.385
Triglyceride (mmol/L)	0.93(0.37)	0.91(0.32)	1.03(0.41)	5.925	0.052
HDL-C (mmol/L)	1.52 ± 0.33	1.65 ± 0.39	1.73 ± 0.44	3.768	0.025
LDL-C (mmol/L)	2.98 ± 0.93	2.89 ± 0.94	3.01 ± 0.96	0.244	0.784
HDL-C/LDL-C	0.66 ± 0.89	0.67 ± 0.34	0.68 ± 0.33	0.010	0.990
Female/n	117	179	43	-	-
Age (years)	94.07 ± 3.30	92.99 ± 2.86	93.65 ± 3.28	4.432	0.013
Body mass index (kg/m^2^)	19.86 ± 3.25	19.89 ± 3.24	21.44 ± 4.25	3.970	0.020
Systolic blood pressure (mmHg)	167.16 ± 27.44	167.87 ± 29.84	168.47 ± 28.78	0.038	0.962
Diastolic blood pressure (mmHg)	91.08 ± 13.38	88.04 ± 13.40	91.84 ± 13.21	2.526	0.082
Total cholesterol (mmol/L)	5.27 ± 0.94	5.16 ± 0.98	5.10 ± 0.96	0.645	0.525
Triglyceride (mmol/L)	1.09(0.76)	1.00(0.51)	0.95(0.41)	2.923	0.232
HDL-C (mmol/L)	1.55 ± 0.30	1.62 ± 0.37	1.64 ± 0.30	1.603	0.203
LDL-C (mmol/L)	3.19 ± 0.77	3.07 ± 0.86	2.97 ± 0.84	1.255	0.287
HDL-C/LDL-C	0.57 ± 0.23	0.58 ± 0.29	0.66 ± 0.29	2.954	0.053

HDL-C, high-density lipoprotein cholesterol; LDL-C, low-density lipoprotein cholesterol. The value of triglyceride was presented as median (interquartile range), the difference among the genotypes was determined by the Kruskal-Wallis Test.

**Table 6:** Comparison of clinical characteristics and serum lipid levels among genotypes stratified by gender in non-LG

Parameter	B1B1	B1B2	B2B2	*F*	*P*
Male/n	96	133	24	-	-
Age (years)	53.04 ± 8.00	52.21 ± 8.95	47.58 ± 7.11	4.044	0.019
Body mass index (kg/m^2^)	21.96 ± 2.99	22.46 ± 3.25	22.00 ± 2.62	0.808	0.447
Systolic blood pressure (mmHg)	133.63 ± 18.41	128.77 ± 18.25	129.42 ± 18.40	2.022	0.135
Diastolic blood pressure (mmHg)	88.88 ± 11.99	83.68 ± 9.89	87.42 ± 15.63	6.040	0.003
Total cholesterol (mmol/L)	4.90 ± 1.11	4.82 ± 0.94	5.43 ± 1.21	3.531	0.031
Triglyceride (mmol/L)	1.22(0.76)	1.16(1.02)	1.28(1.38)	3.231	0.199
HDL-C (mmol/L)	1.44 ± 0.27	1.52 ± 0.27	1.69 ± 0.27	8.180	0.000
LDL-C (mmol/L)	2.76 ± 0.99	2.63 ± 0.93	2.71 ± 0.90	0.485	0.616
HDL-C/LDL-C	0.75 ± 1.16	0.68 ± 0.37	0.72 ± 0.34	0.196	0.822
Female/n	110	107	28	-	-
Age (years)	52.51 ± 9.27	54.95 ± 8.56	57.93 ± 8.97	4.792	0.009
Body mass index (kg/m^2^)	22.71 ± 5.18	21.45 ± 3.18	21.82 ± 2.70	2.562	0.079
Systolic blood pressure (mmHg)	132.62 ± 19.22	131.21 ± 24.28	132.21 ± 23.53	0.112	0.894
Diastolic blood pressure (mmHg)	85.09 ± 10.59	84.15 ± 12.22	83.00 ± 10.15	0.446	0.641
Total cholesterol (mmol/L)	4.69 ± 0.79	5.05 ± 0.92	4.55 ± 0.80	6.635	0.002
Triglyceride (mmol/L)	1.01(0.95)	0.98(0.45)	1.18(1.41)	0.975	0.614
HDL-C (mmol/L)	1.45 ± 0.30	1.54 ± 0.30	1.60 ± 0.28	3.788	0.024
LDL-C (mmol/L)	2.54 ± 0.80	2.93 ± 0.84	2.38 ± 0.70	8.591	0.000
HDL-C/LDL-C	0.64 ± 0.28	0.60 ± 0.35	0.74 ± 0.33	2.270	0.106

HDL-C, high-density lipoprotein cholesterol; LDL-C, low-density lipoprotein cholesterol. The value of triglyceride was presented as median (interquartile range), the difference among the genotypes was determined by the Kruskal-Wallis Test.

### Correlation between serum lipid parameters and genotypes

Multiple linear regression analysis showed that serum TG and HDL-C levels and HDL-C/LDL-C ratio were correlated with genotypes in LG, whereas serum TC and HDL-C levels were associated with genotypes in non-LG (*P *< 0.05-0.001; Table 7).

**Table 7:** Correlation between serum lipid parameters and the CETP *Taq*IB polymorphism in LG and non-LG

Lipid	Relative factor	Unstandardized coefficient	Standard error	Standardized coefficient	*t*	*P*
LG plus non-LG						
HDL-C	Genotype	0.080	0.015	0.160	5.316	0.000
LG						
TG	Genotype	-0.064	0.029	-0.093	-2.191	0.029
HDL-C	Genotype	0.074	0.023	0.138	3.219	0.001
HDL-C/LDL-C	Genotype	0.054	0.016	0.149	3.464	0.001
Non-LG						
TC	Genotype	0.132	0.065	0.089	2.030	0.043
HDL-C	Genotype	0.084	0.019	0.189	4.435	0.000

## Discussion

*Taq*IB is one of the common polymorphisms of the CETP gene. In the current study, the overall frequency of B2 allele was 0.372 (0.384 in LG and 0.345 in non-LG), similar to our previous finding in Hei Yi Zhuang, another Zhuang subgroup resides in Napo, a county bordering northwest Vietnam [34,36], and to the result from different populations, such as Han Chinese (0.39), Vietnamese (0.34), and Korean (0.36) [37-39], but lower than that in Caucasians (0.40-0.64) [14,40], Jewishes and Japaneses [17,41,42]. The considerable high frequency of B2 mutation across populations worldwide appears to imply that the origin of B2 can at least be traced back long before the differentiation of major ethnic groups for some selective reasons. We did not observe significant difference in genotypic and allelic frequencies of CETP *Taq*IB polymorphism between the two groups; this was in agreement with those of the Japanese centenarian study [13,17]. However, there was a trend that the frequencies of B1 allele and B1B1 genotype in long-lived individuals was slightly lower than those in non-long-lived subjects, or conversely, the B2 allele tended to enrich in the elderly old. This observation seems to imply that the evolution of B2 allele may be favorable for the survivals of modern human beings whose living environment and dietary structure have been changing tremendously, at least in Bama area.

It has been established that HDL-C is an independent risk factor for CHD [43]. A growing body of evidences demonstrate that elevated levels of HDL-C may reduce CHD risk and thus contribute to longer life expectancy [13]. We also found higher HDL-C and LDL-C levels in the long-lived subjects as compared to general populations. Moreover, this raised HDL-C was associated with B2 allele and B2B2 genotype, which was consistent with some previous reports [12,14,34,44-46], but not others [17,47]. It is widely accepted that B2 allele carriers exhibit lower CETP levels and/or activity, leading to a dysfunction of the reverse cholesterol transport, causing an accumulation of cholesterol ester in HDL style and thus increasing the levels of HDL-C. The mechanism by which *Taq*IB polymorphism may affect CETP activity or HDL-C levels is not well known. Because this polymorphism located in an intron of the CETP gene which is unlikely to exhibit any functional effect. Given the reported associations of the B2 allele with increased CETP mass and/or activity, the most putative explanation is that this polymorphism is in linkage disequilibrium with some unknown functional mutations in the regulatory region of the CETP gene which might interact with each other to determine the HDL-C levels [45,48].

Some investigators suggested that *Taq*IB polymorphisms were solely associated with HDL-C levels, without being influenced by factors such as smoking, alcohol consumption and BMI [49], but most researchers believed that the association between *Taq*IB polymorphisms and HDL-C levels might be greatly affected by dietary habits, smoking, obesity, gender, and ethnic groups, etc. [34,50-52]. In the present study, we also showed that the association of CETP *Taq*IB polymorphism and serum lipid profiles is different between LG and non-LG, or between males and females in the Chinese Bama Zhuang population. The subjects with B2B2 and B1B2 genotyes in LG had higher HDL-C levels and HDL-C/LDL-C ratio than the subjects with B1B1 genotye, whereas the B2 allele carriers in non-LG had lower TC and higher HDL-C levels than the B2 allele noncarriers. The levels of HDL-C in LG were different among the genotypes in males but not in females. The levels of HDL-C in non-LG were different among the genotypes in males, whereas the levels of TC, HDL-C and LDL-C were different among the genotypes in females. The reason for this discrepancy is not well known. Thus, to better define the exact association of *Taq*IB polymorphisms with serum HDL-C levels and CHD, further studies are required in large populations.

## Conclusion

The current study shows that the genotypic and allelic frequencies of CETP *Taq*IB polymorphism were not different between LG and non-LG, but the association of CETP *Taq*IB polymorphism and serum lipid profiles is different between the two groups in the Chinese Bama Zhuang population. The B2 allele and B2B2 genotype were associated with high HDL-C levels. Thus, CETP *Taq*IB polymorphism might be one of the longevity-related genes in this population.

## Competing interests

The authors declare that they have no competing interests.

## Authors' contributions

SLP participated in the design, undertook genotyping, and drafted the manuscript. FW, CWL, JHP, XQL, SHL, HYW and LJH helped with genotyping. ZPL, CYH, HL and GFP took part in the epidemiological survey and collected the samples. RXY conceived the study, participated in the design, carried out the epidemiological survey, collected the samples, and helped to draft the manuscript. All authors read and approved the final manuscript.
